# Design and Evaluation of Volunteer User Trials of Unobtrusive Vital Signs Monitoring for Older People in Care Using Wi-Fi CSI Sensing

**DOI:** 10.1109/JTEHM.2025.3624469

**Published:** 2025-10-22

**Authors:** Aaesha Alzaabi, Imran Saied, Tughrul Arslan

**Affiliations:** Institute for Integrated Micro and Nano SystemsThe University of Edinburgh, Scotland 3124 Edinburgh EH9 3FF U.K.; Advanced Care Research CentreUsher Institute, University of Edinburgh 3124 Edinburgh EH8 9AG U.K.

**Keywords:** Care technology, channel state information (CSI) sensing, non-contact sensing, user trials

## Abstract

Objective: This study describes the design and evaluation of volunteer user trials of an unobtrusive Wi-Fi Channel State Information (CSI) vital sign sensing system in older participants aged 60 years and older in different home environments. Methods and procedures: In terms of experiment design, the implementation of user-centric sensor placement and integration informed consent with various experimental elements in the design of experiments of older people. The implemented signal processing algorithm, which extracts vital signs from the Wi-Fi CSI signal to obtain respiration and heart rate measurements, employs wavelet filtering techniques. For selecting of vital sign signals from the 52 CSI subcarriers, the Principal Component Sample Entropy (PC-SampEn) was implemented to capture the information most relevant to vital signs.Results: Two cardiorespiratory vital sign measurements were validated against wearable ground-truth devices, a respiratory belt and a photoplethysmogram (PPG). The results demonstrated an expected decrease in accuracy and measurement agreement in uncontrolled home environments.Conclusion: Although respiratory rate measurements have demonstrated promising accuracy and agreement in uncontrolled environments, heart rate measurements observed high variability in these scenarios due to challenging signal extraction. Further experiments must be conducted to address the limitation in sample size and the technical challenges in heart rate signal extraction to improve accuracy. Clinical and Translational Impact: This study provides a design of unobtrusive care technology for vital sign sensing for older adults, demonstrated and evaluated in the context of in-home monitoring for healthcare.

## I. Introduction

The increase in life expectancy in most developed countries, including the United Kingdom, presents several challenges to the health system regarding the care of older people [1], [2]. The decline in birth rates in developed countries, coupled with this increase in life expectancy, contributes to the shifting of the population’s age structure towards the older ages [2], [3]. Older adults experience a high prevalence of chronic conditions, such as cardiovascular disease, asthma, and stroke. Due to improved medical care and early diagnoses, older individuals are living longer with disabilities and chronic conditions, such as cardiovascular diseases [1]. This necessitates the development of innovative and sustainable solutions for continuous health monitoring and incident detection. From a healthy ageing perspective, there is a growing need to facilitate such solutions for older individuals ageing-in-place, thereby maintaining their quality of life and prioritizing their autonomy for longer.

Vital signs reflect the most crucial body functions, namely parameters such as respiratory rate (RR), heart rate (HR), temperature, and blood pressure. The evaluation of a person’s vital signs provides valuable information about their overall health and aids in identifying possible underlying conditions. Among the aforementioned vital signs, RR and HR have been found to be reliable indicators of cardiopulmonary arrest [4]. Despite being the most neglected vital sign, abnormal RR remains the most important predictor of cardiac arrest and unplanned admission to the intensive care unit (ICU) [5]. Furthermore, changes in RR and HR are associated with the risk of falls in older adults. Survey data and analyses identify the occurence of falls or the risk of falls affect 40% to 60% of adults with cardiovascular disease [6]. In addition, postoperative respiratory depression can occur in patients recovering from surgery and anaesthesia, leading to cardiorespiratory arrest and increased mortality; therefore, the monitoring of respiratory activity is necessary to facilitate timely intervention [7]. Continuous monitoring of vital signs has been shown to be more effective in capturing adverse events than manual measurements performed during nursing rounds in a medical setting [8].

In order to provide patient-centred care, the design of unobtrusive at-home sensing solutions must take into account human factors, such as the pReferences of older people to age-in-place and maintain their independence [9], [10]. Patient perspectives play a vital role in the effective design of long-term monitoring devices. Studies have shown that wearable health monitoring devices can be intrusive, especially during periods of illness, which in turn influences long-term adherence [9], [11]. As wearable devices often encounter problems of ‘remembrance, rebellion, and obtrusiveness’ among the older population, unobtrusive sensors could alternatively be embedded in the home environment to become ambient [12], [13]. This approach would enhance patient compliance and data continuity, potentially contributing to the early prediction of health disorders for clinical use.

Unobtrusive smart sensor systems could enhance the quality of care for older patients by automating routine tasks, such as vital sign measurements, thereby allowing care providers to focus on more essential aspects of patient health. The ability of these systems to provide continuous measurements could facilitate care providers in the assessment of overall health and detection of incidents that require immediate attention. Unobtrusive health monitoring is defined as the use of ambient sensor technology to acquire human health-related data without causing inconvenience to daily life [14]. In this work, we introduce user trials of our non-contact Wi-Fi Channel State Information (CSI) sensor system which captures RR and HR unobtrusively through dynamic signal reflections caused by body movements due to cardiorespiratory activity. Despite the widely researched applications of unobtrusive Wi-Fi CSI sensing, reports on user trials involving this technology within the older population remain limited.

User trials are a fundamental component in the development and validation of health technologies, as they allow for a realistic assessment of the usability of the proposed system. Additionally, they encourage bridging the gap between theoretical research and practical implementation through the lens of iterative experimental and system design. Evaluating prototypes in the homes of older adult volunteers provides invaluable feedback to optimise the system by identifying barriers to implementation and system usability. Considering the end-user in context at various stages of the study design, from ideation to assessment of prototypes, is essential to ensure technology compatibility with user needs. For instance, calibrating the system algorithms may be necessary to adapt to variations in physiology and environment.

This study describes the design and evaluation of volunteer user trials of our unobtrusive Wi-Fi CSI vital sign sensing system in older adults residing in different home environments. This work is part of an ongoing research project within the Integrated Technologies of Care work package at the Advanced Care Research Centre (ACRC) at the University of Edinburgh [15]. The ACRC is a multi-disciplinary research programme integrating research across fields including medicine and other care professions, engineering, informatics, data and social sciences [16]. Drawing on the expertise from this collaborative framework, we initiated the evaluation of our unobtrusive sensing system in real-world home settings. The contributions of this study are as follows:

• The implementation of user trials in older adults’ homes and the living lab while incorporating informed consent in the data collection process for each subcomponent. Older adults who participated in our study, aged 60 years or older, were instructed to sit or rest as they usually would in their home environment. Subsequently, our unobtrusive sensors were integrated into their living spaces without requiring any rearrangement of their furniture. Volunteers also had the option to allow the collection of ground-truth vital sign data from a respiratory belt and a photoplethysmograph (PPG), depending on their comfort level with the measurements being taken. While the development of ambient sensor technology has been extensively investigated in the current literature [17], [18], [19], the implementation of user trials for older individuals in home settings remains limited.
• The signal processing procedure employs an enhanced signal extraction method to isolate vital signs from high-dimensional Wi-Fi CSI data. In it, Principal Component Analysis (PCA) and Sample Entropy (SampEn) are combined to identify the signal with maximum variance and regularity. While PCA reduces dimensionality and captures global signal variance, SampEn assesses the complexity of the resulting principal components (PCs). The PC with the lowest SampEn is then selected as the signal containing the most relevant information about the RR and HR. This complementary strategy effectively captures vital sign information that is irrelevant to environmental variations in older persons’ homes. Although previous studies have reported on their system performance in controlled lab environments or in a single home location [17], [18], our approach evaluates system performance in various home settings of older adults.

## II. Related Works

Unobtrusive sensing technologies present a wide range of promising applications for the care of older adults, while providing the benefits of continuous data collection and enhanced user acceptance compared to contact-based counterparts [14]. Typically, contact-based sensors, particularly those that rely on single-point contact, can limit the amount and type of information collected due to the location of the sensor [20]. Additionally, they may interfere with older adults’ comfort-level during the day and impact their sleep quality. Wearable sensors, especially those requiring direct skin contact, can cause skin irritation or cross-infection if shared by multiple users [21]. In contrast, unobtrusive sensors can be seamlessly integrated into daily environments without disrupting older adults’ daily routines or requiring them to adhere to the wearing of monitoring devices. As a result, user health can be continuously monitored, allowing the early detection of physiological abnormalities and thereby enhancing clinical outcomes [22].

The contactless vital sign sensing techniques reported in the literature utilize a wide range of sensors from video and thermal cameras [23], visible light sensors [24], microphones [25], [26], to Radio Frequency (RF) sensing [27], [28], [29], [30]. Although older adults generally display positive attitudes towards unobtrusive sensing, modalities based on video cameras often raise privacy concerns [31], [32], [33]. Additionally, video cameras and visible light sensors tend to be susceptible to changes in lighting conditions, physical occlusions, and variations in subjects’ skin colour [23], [34].

There has been a growing interest in utilizing non-contact RF-based technologies for monitoring physiological parameters in healthcare applications. These RF-based sensors can detect vital signs and human activities despite occlusions, while ensuring the user’s personal privacy [35]. RF-based sensing encompasses different radar modalities, such as Doppler Continuous Wave (CW) [36], Frequency Modulated Continuous Wave (FMCW) [37], and Impulse Radio Ultra Wide Band (UWB-IR) [38], [39], as well as Wi-Fi-based techniques using Received Signal Strength Indicator (RSSI) [40] and CSI [28], [41]. Although radars can accurately measure vital signs through walls and various materials, they require specialized hardware which can often be costly for large-scale deployment.

On the other hand, Wi-Fi-based sensing can leverage existing wireless infrastructure and affordable off-the-shelf components due to its widespread use and ubiquity in consumer electronics [42]. Earlier studies on Wi-Fi sensing have utilized RSSI to track human activities [43] as well as RR [40]. While Wi-Fi RSSI is coarse-grained [44], Wi-Fi CSI can provide fine-grained information about the wireless channel, allowing for a more detailed identification of the multipath characteristics [45].

In recent years, Wi-Fi CSI sensing has been investigated in the literature for various motion-based physiological monitoring applications such as RR and HR [46], [47], blood pressure [48], activity monitoring [49], [50], sleep monitoring [51], [52], fall detection [53], [54] and gait analysis [55], [56]. In terms of sensing device acceptability, it should be investigated in a representative cohort to obtain an unbiased assessment of its applicability and performance. However, there is a notably limited account of user trials for Wi-Fi CSI unobtrusive vital sign sensing for older people, aged 60 and over, despite the relevance of its application.

In Wi-Breath [57], the authors develop an RR monitoring algorithm based on CSI amplitude and phase difference, utilizing a Support Vector Machine algorithm for optimal signal selection. However, the study was limited to a small number of participants. Meanwhile, in [58], an HR monitoring method is proposed using Wi-Fi amplitude and phase information, alongside a frequency domain subcarrier selection algorithm based on the heartbeat-to-subcomponent ratio. The study collected data from nine participants over the course of a month in two different environments. Considering studies that gather both RR and HR, the authors in [17] propose a contactless Wi-Fi CSI monitoring system, based on CSI phase difference, where the experiments were carried out for three months with four volunteers in a laboratory environment.

In larger-scale studies, such as in [18], a Wi-Fi CSI monitoring system is presented for respiratory rate and patterns using CSI ratio and multiple antennas, for 21 participants in a controlled laboratory environment, aged between 14 and 57 years. MoBreath [19] uses Wi-Fi CSI from commodity smartphones in different environments on 10 research participants, most of whom were under 30 years old, with only one participant being 60 years old. As far as we know, most of the current CSI-based vital sign monitoring studies reported their implementations in a small number of mostly younger adult participants in controlled environments, highlighting the need for technology validation in older adults in their home settings. This research endeavors to address the technical challenges of in-home deployment by conducting short-term user trials in older adult volunteers’ homes aimed at extracting cardiorespiratory parameters.

## III. Methodology

This exploratory pilot study is part of a larger project focused on developing systems for unobtrusive health monitoring of older people within the context of care. Within the scope of this initiative, this study aims to enhance the study design for conducting user trials with older adults in their daily life settings, prior to scaling up to long-term deployment and to a larger sample size. The insights gained from this preliminary exploration will inform subsequent phases of this research project, specificaly in refining data collection methods and study protocols aimed at unobtrusive health monitoring for older people for care.

### A. Ideation and Co-Design of Sensing Solutions

In the initial stages of the project, particularly during the ideation phase, stakeholder input played a significant role in the development of our sensing system for care, as it incorporates user experience and needs into the design process [33]. The primary stakeholders involved in this phase included 19 clinicians, healthcare professionals, and members of the public. The continuous stakeholder engagement from the early stages of the study aligns technology with real-world needs by influencing sensor design priorities and establishing a robust foundation for effective long-term deployment.

Stakeholder involvement informed the user needs that are to be addressed by the sensor design, as well as the selection of key health parameters for the care of older people. This preliminary study concluded that vital signs, hydration, and movement are the most critical parameters to extract, with non-contact sensing modalities prioritized to preserve older individuals’ dignity and privacy [33]. With feedback from stakeholders, we developed several unobtrusive sensors and validated them in a controlled laboratory environment [59], [60], [61].

In the second phase, a Patient and Public Involvement (PPI) workshop was held to facilitate discussions on unobtrusive technologies. It was held among 5 researchers and 9 members of the public who participated in assessing the sensors in application [33]. The sensors under development were demonstrated before breaking out into moderated discussion groups. The main goal of the discussion was to evaluate the acceptability and feasibility of unobtrusive sensing with members of the public, in order to co-design the subsequent stages of sensor development. Although live demonstrations and PPI workshops provided valuable information for the iterative design of unobtrusive sensors for care, some barriers to implementation can only be identified through volunteer testing in real home environments.

### B. Volunteer Recruitment

The participants for the first round of this study were recruited over a 10-month period, from May 2024 to February 2025. The target population of this study consists of older adults aged 60 years or older living independently. The recruitment strategy for the study included the distribution of brochures in public libraries and community centers, as well as the organization of information sessions in community clubs. The inclusion criteria for this study required that participants be 60 years or older, live independently, and be capable of giving informed consent. The exclusion criteria were the presence of any severe mobility or respiratory problems during the planned experiment, and inability to give informed consent. All materials and procedures were approved by the Ethics Committee of the School of Engineering at the University of Edinburgh. According to the UK’s Medicines and Healthcare products Regulatory Agency (MHRA) tool, the outcome indicated that this study is considered research and does not require a National Health Service (NHS) Research Ethics Committee review in Scotland.

During the recruitment process, a total of 20 potential volunteers were approached and pre-screened according to the inclusion and exclusion criteria. After being approved to volunteer for the study, the researchers visited potential participants’ homes with the required sensors and materials. Upon arriving at their home location, brief introductions to the experiments are provided, while the potential participants received a participant information sheet detailing the procedure and answering common questions. The participant information sheet contained information such as how the data would be processed and stored, who would have access to it, and their right to withdraw from the study. In total, 12 individuals signed the consent forms to participate in the study after being given enough time to ask questions. Of the 12 study participants who consented to participate in the unobtrusive sensor experiments, only seven participants were willing to wear the respiratory belt and heart rate monitor ground-truth devices for paired measurements.

**FIGURE 1.**
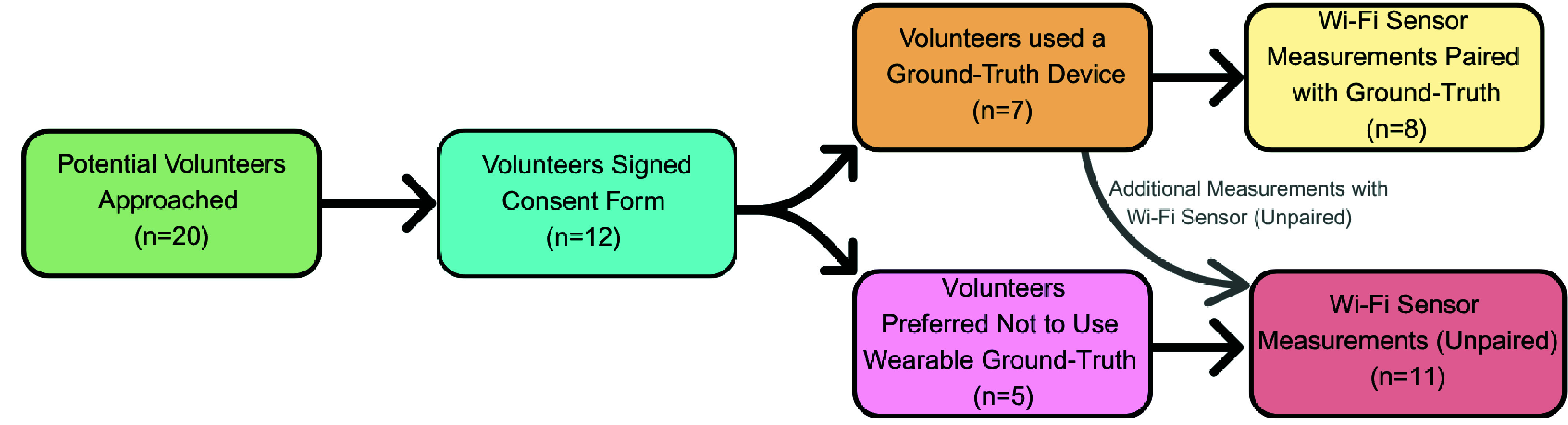
Older people’s (60 years or older) participation as study volunteers for the unobtrusive vital sign measurement sensor implementation in their home. While some of the volunteers preferred not to use the ground-truth wearable sensors for various reasons, additional measurements were taken if time permitted.

Regarding the study participation and additional measurements, five of the study participants provided additional readings which were both paired and unpaired with the ground truth. However, another five participants did not feel comfortable using wearable devices and did not have the paired readings taken using them. Each component of this study, including the location of the setup and the sensors included, was designed to be entirely voluntary to ensure the comfort and well-being of the participants. A diagram showing the participation of older people in the study is shown in Fig. 1.

### C. System Implementation

To capture vital parameters for older adults unobtrusively, we developed an unobtrusive vital sign sensing system using the ESP32, an affordable off-the-shelf (OTS) microcontroller unit (MCU). Specifically, utilizing two Espressif ESP32-DevKitC-VE devices [62], one configured as a transmitter (TX) and the other as a receiver (RX) operating in infrastructure mode. The transceivers utilize the built-in omnidirectional printed circuit board (PCB) meandered planar inverted f antenna on the off-the-shelf development kit. The Wi-Fi used by the ESP32 MCU operates at 2.4 GHz with a maximum Effective Isotropic Radiated Power (EIRP) of 20 dBm, in accordance with the IEEE 802.11n and European Telecommunications Standards Institute (ETSI) standards. The ESP32 is enclosed in a 3D-printed wall-mountable case made using a Polyethylene Terephthalate Glycol (PETG) material to make it more aesthetically acceptable for residential homes.

The ESP32-CSI-tool [63] was used to access and collect Wi-Fi CSI data from the ESP32 MCU. The baud rate for serial communication speed was configured at 1843200 bits per second, and the data was collected in.CSV format using a macOS laptop. Each received CSI data was time-stamped with Unix epoch time to prepare it for post-processing. The transmit rate was selected as 120 packets per second (PPS) to capture vital signs, which suffices considering that an older adult ’s maximum resting heart rate is 100 beats per minute, equivalent to 1.67 Hz [64].

The NUL-236 Respiration Belt logger [65] and the NUL-208 Heart Rate and Pulse logger [66] sensors were used to obtain ground-truth measurements for RR and HR, respectively. The Respiration Belt Logger is a sensor strapped around the participant’s chest to measure the variation of the air pressure within the belt. The air pressure measurements from the belt are recorded in arbitrary units, ranging from 0 to $20,000$ with an Analogue-to-Digital Converter (ADC) resolution of 15 bits and a maximum sampling rate of 100 samples per second. The corresponding RR can be calculated from the resulting waveform acquired from the belt. To accommodate different body shapes and sizes, an extension velcro strap was provided with the respiratory belt. Meanwhile the Heart Rate and Pulse Logger is a PPG which can be attached to the participant’s finger or earlobe, capturing HR ranges from 0 to 240 BPM, with a measurement uncertainty of $\pm 1 BPM$, with a maximum sampling rate of 100 samples per second. The accompanying Neulog software application was used to collect ground-truth RR and HR measurements at a sampling frequency of 50 Hz, saving the values in a.CSV file format.

### D. From the Lab to the Home

The experiments conducted in this study primarily took place in volunteer homes; however, the volunteers were also given the option of conducting the experiments in the labratory setting, if this was more convenient for them. Three out of the twelve volunteers had their measurements taken in the labratory, one of whom had ground-truth measurements recorded for two readings. For the remaining nine volunteers, six consented to have ground-truth measurements taken using wearables in their homes. To facilitate for the most natural breathing behavior, meditation music was played in the background during most experiments, and the recorded intervals were captured at random instances. Implementing this approach has helped capture a more natural breathing behaviour by alleviating the stress associated with being observed while taking breathing measurements, mitigating the Hawthorne Effect.

**FIGURE 2.**
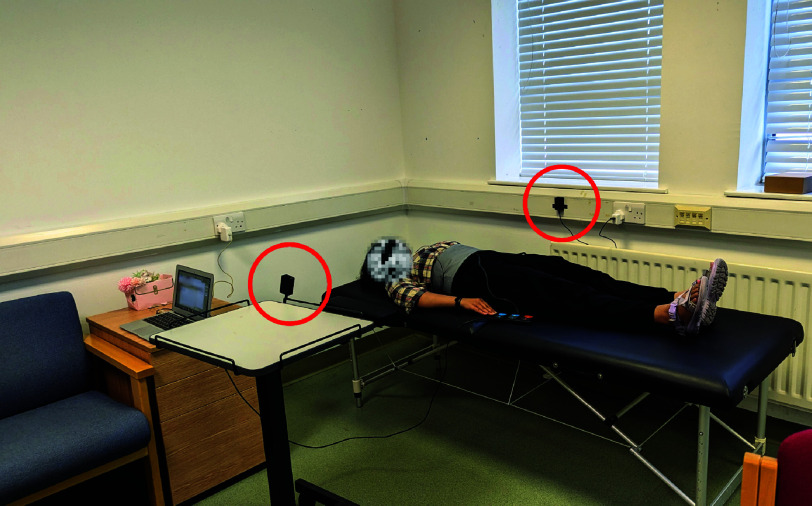
The unobtrusive sensing living lab of the ACRC’s Integrated Technologies of Care at the University of Edinburgh is offered as an optional setup. The sensors can be placed bedside (circled in red) if the volunteer prefers to lie down, or on the wall if they prefer to sit in a chair. The Wi-Fi CSI sensor is pictured collecting raw data for RR and HR extraction, paired with a ground-truth sensor.

The laboratory is located in the Living Lab of the Integrated Technologies of Care of the ACRC at the Scottish Microelectronics Centre of the University of Edinburgh. As shown in Fig. 2, the labrotory simulates a one-bedroom studio apartment environment with includes a bed, bedside table, chairs, coffee table, television, and kettle. For safety and comfort measures, volunteers were given the option to choose between two different bed heights, as well as a low armchair with an orthopedic booster cushions to adjust for height.

**FIGURE 3.**
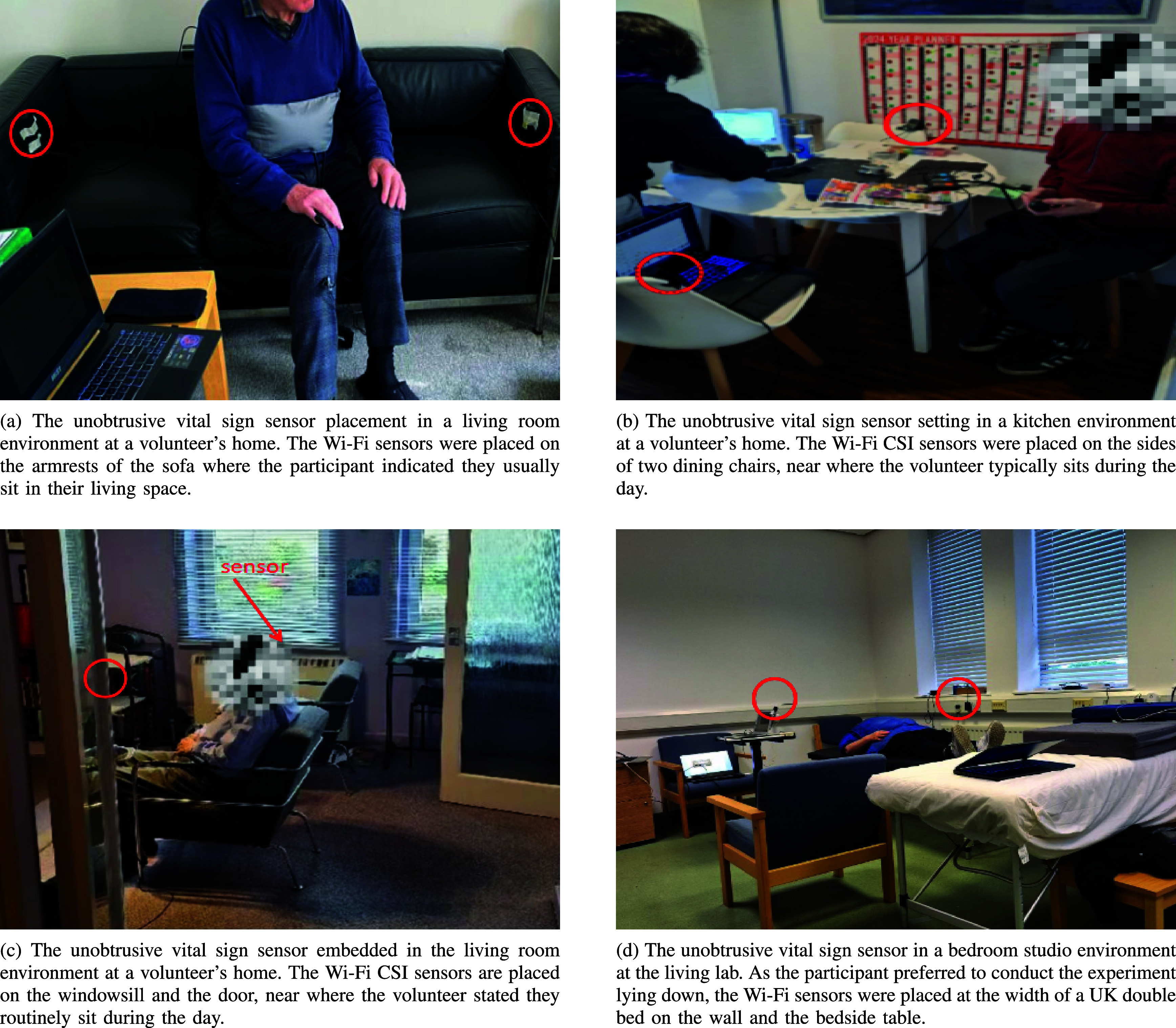
Sample photos illustrating some of the various sensor configurations implemented during real-world user trials of the Wi-Fi CSI vital sign measurement sensing system. The sensor locations and distances varied across different volunteers depending on their daily routines, personal preferences, and the specifics of the location.

The implementation of the system in volunteers’ homes accommodates to the environmental characteristics of their living spaces. We inquire about the participants’ regular seating location in their living space, where they spend most of their day, and adjusted the physical installation accordingly. Factors such as pre-existing furniture placement and power outlet locations were considered before determining the optimal placement of the preconfigured sensing system components in the participants’ home. Items such as Universal Serial Bus (USB) cables of various lengths, extension cords, tripods, and wall-safe tape were used to accommodate different room configurations. Fig. 3 demonstrates various ways in which the unobtrusive Wi-Fi sensor has been embedded in living environments.

### E. Signal Processing and Vital Sign Extraction

**FIGURE 4.**
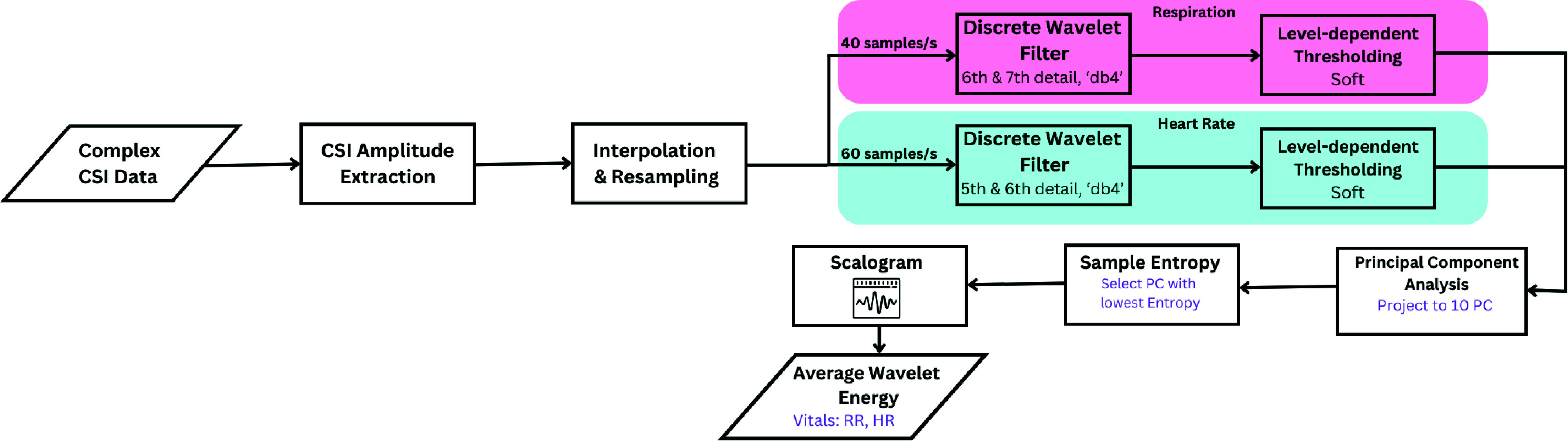
The signal processing flow for the extraction of vital signs from the complex Wi-Fi CSI data. From the same CSI amplitude signal, the RR (as highlighted in light red) and HR (as highlighted in light blue) are resampled and filtered separately. The proposed PC-SampEn is implemented on the filtered signals to extract the most relevant components reflecting cardiorespiratory activity.

The goal of using a non-contact Wi-Fi CSI sensor system is to detect the dynamic changes in the RF signal reflections caused by human cardiorespiratory body motions. The expected chest displacement due to respiratory motion is between 4 to 12 mm and between 0.2 to 0.5 mm for heart rate [67]. The subtle body movements due to respiratory and cardiac activity induce signal variations in the dynamic path of the CSI, which can be leveraged to extract vital sign parameters. Nonetheless, due to hardware imperfections and RF signal propagation, various sources of systematic error could arise, such as packet propagation delays, carrier frequency offset, and sampling frequency offset. After signal acquisition, as detailed in Section III-C, the signal processing procedure illustrated in Fig. 4 is implemented to extract the RR and HR parameters.

The vital sign signal extraction process begins by first obtaining the CSI amplitude from the complex CSI data, such that:

\begin{equation*} |CSI(t_{n})|_{n=1}^{N}=\sqrt {CSI_{Re}(t_{n})^{2}+CSI_{Im}(t_{n})^{2}}, \tag {1}\end{equation*}

where $t_{n}$ is the observed time of the $n^{th}$ received packet for $N$ total number of packets, and $CSI_{Re}$ and $CSI_{Im}$ are the real and imaginary components, respectively.

The received Wi-Fi CSI packets are not uniformly interleaved in time due to the randomness of packet arrival times and propagation delays. To achieve uniform data spacing, the CSI data is interpolated and resampled using the Fourier Method. The interpolation and resampling processes facilitate the removal of outliers and the even distribution of signal samples in time to prepare them for the subsequent signal processing steps. Since the Wi-Fi packet transmission rate is set to 120 PPS, the signal is subsequently downsampled to 40 PPS for RR and 60 PPS for HR. The signal processing flow is illustrated in 4, where the RR and HR extraction process is highlighted in light red and light blue, respectively.

The Discrete Wavelet Transform (DWT) Filter has been chosen due to its ability to handle waveforms of non-stationary nature, such as physiological signals. According to a previous study by Wang et al. [17], the impact of different mother wavelets has been evaluated. The findings reveal that the ‘db4’ to ‘db7’ wavelets achieve comparable performance in RR and HR estimation using Wi-Fi CSI. The fourth-order Daubechies orthogonal wavelet ‘db4’ is selected as it optimally corresponds to the signal characteristics of respiration and heart movement signals while minimizing the number of filter taps. This selection reduces the effect of outliers while minimizing computational cost [17], [68].

The known frequency bands for each vital sign parameter guide our selection of decomposition levels, where the preserved coefficient levels contain the desired frequencies. For older adults living independently, the resting RR typically ranges from 12 to 18 Breaths Per Minute (BrPM) and may increase up to 25 BrPM for those in long-term care settings. We select the range of [9, 37] BrPM to include RRs associated with dyspnoea and tachypnoea, corresponding to the sixth and seventh detail coefficients at a sampling frequency of 40 Hz. Meanwhile, the frequency range selected for HR is [28.125, 112.5] Beats Per Minute (BPM), corresponding to the fifth and sixth detail coefficients at 60 Hz sampling frequency. This range contains the expected HR for older adults at rest from [50,100] BPM. Consequently, the maximum level of decomposition selected for RR is 7 and 6 for HR. In terms of in-band denoising, we apply soft level-dependent thresholding to reduce the effect of the inter-band noise.

For stationary and correlated noise, such as Wi-Fi transmission and hardware noise, level-dependent thresholding can be applied since the variance in the DWT coefficients depends on the decomposition level but is constant within each level [69]. Further details regarding the signal processing framework, including the level-dependent coefficient thresholding approach adopted, were developed and described in our previous work in [59] and [70]. For the RR, the threshold for the sixth and seventh detail coefficients is divided by a factor of 4, while for the HR, the thresholds for the fifth and sixth detail coefficients are divided by factors of 4 and 2, respectively. The implemented approach effectively suppresss the noise while preserving vital sign information relevant to cardiorespiratory motion.

At this stage, the multidimensional time series containing the cardiorespiratory motions consists of 52 CSI streams, reflecting the changes in the CSI dynamic path to varying degrees. PCA can effectively capture the majority of the variance in the CSI data using a few PCs. Moreover, PCA is also effective in filtering out uncorrelated noise. We project each respiratory and heart rate time series onto the first ten uncorrelated PCs.

However, PCA alone is insufficient to fully identify the cardiorespiratory signals from the 52 filtered CSI subcarriers. The choice of PC to be selected to contain the most vital signs and human body movements remains a speculative issue, as highlighted by a survey by Liu et al. [71]. Approximate Entropy (ApEn) and SampEn are commonly used methods for quantifying signal complexity in biomedical time series, in which SampEn shows relative consistency compared to ApEn when varying parameter values [72]. This work proposes the implementation of a signal complexity measure on the PCs, such as the sample entropy (SampEn) to quantify the complexity and irregularity of the resulting PCs. The PC with the lowest SampEn value for the signal can be considered the PC that best represents each physiological parameter. The PC with the lowest SampEn indicates that it contains the most periodic regularity and structure, which is a physiologically relevant characteristic of cardiorespiratory motions. The SampEn is defined as:

\begin{equation*} \mathrm {SampEn}(m,r,N) = -\ln \left ({{\frac {A}{B}}}\right), \tag {2}\end{equation*}

Where $m$ is the embedding dimension, $r$ is the tolerance, and $N$ is the length of the sample. $A$ is the probability that two sequences are similar for $m+1$ points, and $B$ is the probability that two sequences are similar (within the tolerance value $r$) for $m$ points [73]. The distance between the data points is calculated using the Chebychev distance.

**FIGURE 5.**
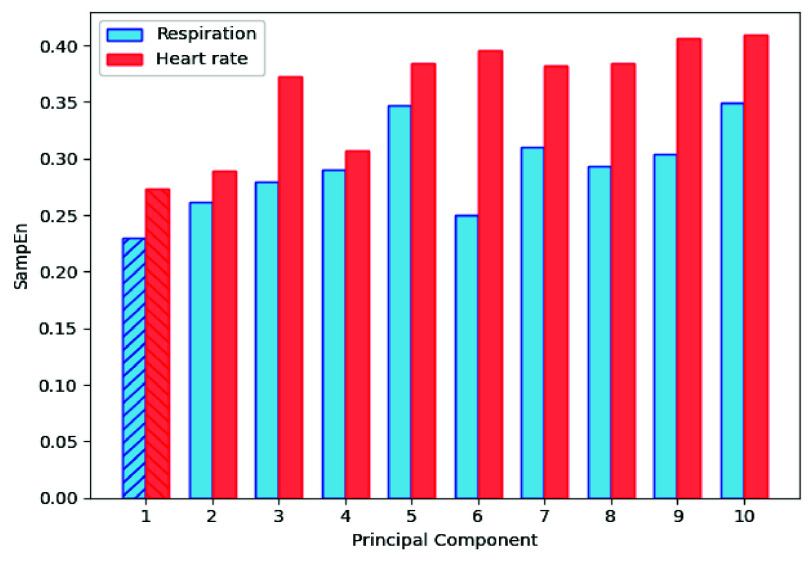
Results of the SampEn applied on 10 PCs for one volunteer, applied to respiration and heart rate data streams.

For our application, we set the embedding dimension as $m=3$ to effectively capture the signal dynamics. The tolerance $r=0.1*\sigma _{x_{PC}}$ is a fraction of the standard deviation of each PC, while $N=F_{s_{down}}*T_{s}$. The SampEn calculations were implemented for each PC using the PyEntropy Python package [74]. The PC with the lowest SampEn value is selected to be the most regular, containing most of the cardiorespiratory signal. A sample result from one of the volunteer trials, with the PC with the lowest SampEn selected is illustrated in Fig. 5.

**FIGURE 6.**
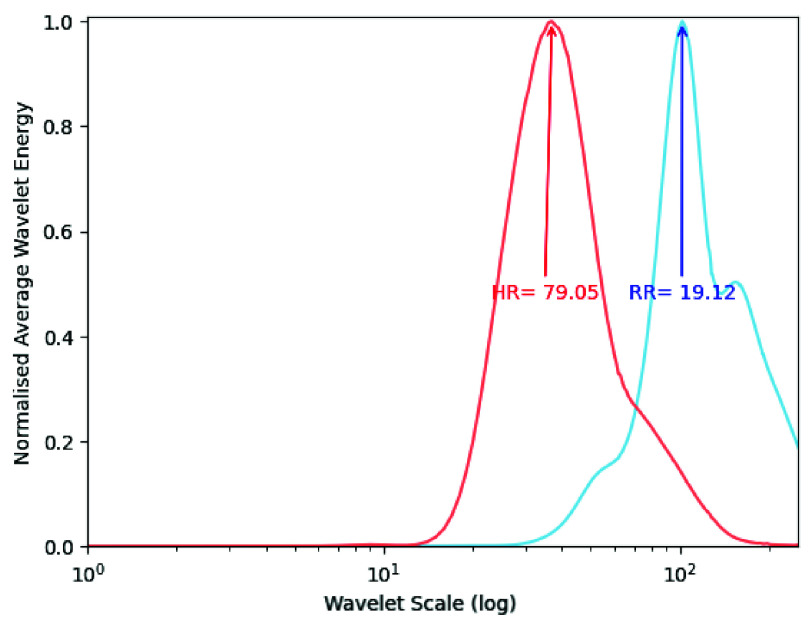
Results of the RR and HR extraction, of one of the volunteers, from the peak of average wavelet energy of the Morelet Scalogram using the ‘db4’ wavelet.

After identifying the PC with the lowest SampEn, the average wavelet energy of the multiresolution scalogram is obtained. The Continuous Wavelet Transform (CWT) is implemented with the Morlet wavelet as a basis function, while using a $2^{8}$ scale. From the resulting scalogram of the chosen PC, the average wavelet energy per resolution level is calculated. The peaks in the average wavelet energy graph correspond to the dominant frequencies in the signal and can therefore be used to estimate RR and HR, as illustrated in Fig. 6 by converting scales to frequency, such as:

\begin{equation*} \mathrm {Rate} = \frac {f_{c}*f_{s}}{a}*60, \tag {3}\end{equation*}

Where $f_{c}$ is the central frequency of the mother wavelet, $f_{s}$ is the sampling frequency of the signal, and $a$ is the wavelet scale. The proposed methodology was validated against the ground-truth using eight measurements from seven older adults across various residential settings. Additionally, 11 measurements were taken from Wi-Fi sensors only. The results of these experiments will be discussed without validation against a ground-truth device. Nonetheless, they will be compared to the sample population averages.

## IV. Results & Discussion

The proposed system was implemented in various home environments with older adult volunteers. Factors including device position and orientation, seating position, furniture placement, clothing material, and environmental clutter all varied across different settings. The system was installed in their home environments, considering their preferred seating during the day and where the device would be placed without impacting their daily routine. Seven out of 12 volunteers have consented to their ground-truth measurements being taken. The time period used for the analysis precluded any major movements at the beginning or end of the recordings. The descriptive statistics of the vital signs are presented and interpreted in this section. Initially, the mean and standard deviation (SD) of the measurements for our Wi-Fi CSI sensing system and ground-truth wearable devices are obtained. The comparison of vital sign measurements between the two devices is accomplished by obtaining the mean absolute error (MAE), root mean squared error (RMSE), Pearson correlation coefficient (PCC), percentage accuracy, as well as the Bland-Altman analysis.

### A. Respiratory Rate Measurements: Results of User Trials

The results of the RR accuracy metrics for the proposed unobtrusive Wi-Fi vital sign sensing system, obtained from eight measurements in seven individuals, are summarized in Table 1.

**TABLE 1:** Wi-Fi sensor RR measurements against ground-truth

	Respiration Belt	Wi-Fi Sensor (RR)
$Mean$	15.31 BrPM	16.14 BrPM
$SD$	3.78 BrPM	5.18 BrPM
$MAE$	1.97 BrPM	
$RMSE$	2.77 BrPM	
$PCC$	0.847	
$Accuracy$	89.25%	

The observed mean and SD of the eight RR measurements from the Wi-Fi sensor are $16.14 \pm 5.18$ BrPM, which differs from the ground-truth by $0.85 \pm 1.40$ BrPM. We note that the MAE of the measurements is 1.97 BrPM, approximately four times higher than the results reported previously in [59] for 47 RR measurements taken under controlled laboratory conditions. Moreover, the standard deviation for 30 repeated measurements is noted to be $\sigma =1.04$ under a controlled condition [59]. Furthermore, the RMSE of the measurements is 2.77, which is nearly 3 times higher than those taken in the controlled laboratory. We also note that the accuracy of the volunteer measurements in home environments is 89.25% compared to the controlled laboratory measurements, which reached up to 96.90%. The PCC between the RR measurements obtained from the ground-truth Respiratory Belt and the Wi-Fi CSI sensor was 0.847, indicating a strong and statistically significant positive correlation.

We attribute various factors to the decreased accuracy and the increased error in the measurements performed in older adults in a home environment compared to a controlled laboratory setting. The previously collected dataset consisted of 47 two-minute measurements on the same individual, with RR controlled by a metronome upon auditory command. In this study, eight measurements were obtained from up to seven individuals with varying respiratory patterns in their home environments, with variable sensor positions, which introduce higher measurement variability. At least two and up to five researchers were present for each in-home experiment, which may have introduced some minor disruptions to the sensor readings. Additionally, the volunteers were advised to relax while the measurements were randomly recorded; during this period, minor body movements were not prohibited. Although these factors may have affected the accuracy of the measurements, they play a crucial role in the experimental design to capture realistic expectations of the accuracy of the RR measurement and to ensure the comfort and safety of our participants.

**FIGURE 7.**
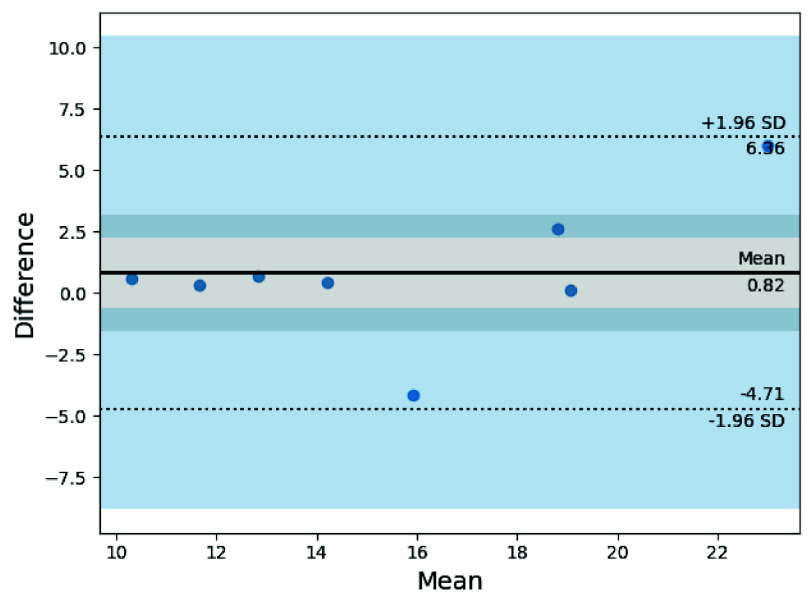
The Bland-Altman mean-difference plot for the Wi-Fi CSI RR measurements against the ground-truth respiratory belt data. The results show signs of proportional bias and heteroscedasticity.

Given the wide range of RR measurement values, we examine the Bland-Altman plot to investigate the measurement agreement between the two instruments. Due to the limited number of measurements, the Bland-Altman plots should be interpreted cautiously. Although the analysis may yield wide limits of agreement, our goal for these preliminary plots is to identify any proportional bias across the captured range of measurements. Healthcare professionals must establish *a priori* the acceptable range or value of inter-rater variability to determine whether the measurement agreement is suitable for in-home health monitoring. Fig. 7 shows the Bland-Altman plot of RR measurements in older volunteers in their home environments.

The Bland-Altman analysis for RR indicates a mean difference of 0.8 BrPM, and limits of agreement of $[-4.71, 6.96]$ BrPM between the Wi-Fi CSI sensing system and the ground-truth respiratory belt. The results suggest a small positive bias, while most of the difference data points lie within $[-0.5,2.5]$ BrPM, reflecting the random measurement error. The RR measurements obtained captured a wide range of expected RRs between 10 and 20 BrPM. Furthermore, we note that the Bland-Altman plot demonstrated some proportional bias across the observed RR measurements, indicating that the mean difference may depend on the RR value. The scatter of the Bland-Altman plot appears to display signs of heteroscedasticity, where the spread of data varies with the mean; however, the results remain inconclusive due to the small sample size. None of the data points falls outside the limits of agreement, within ±1.96 SD, indicating no extreme outliers.

With $n=8$ paired RR measurements, the observed effect size using Cohen’s d was $d= 0.29$. At $\alpha =0.05$ with a two-tailed paired t-test, the study achieved 11.1% power. This indicates that the current study is under-powered, with $n\approx 95$ paired measurements needed to achieve 80% power for this effect size. Although the number of measurements performed in the preliminary round of user trials was limited, these findings provide a framework for validating measurements in volunteer trials and illustrate the potential of unobtrusive Wi-Fi CSI sensing for RR measurements among older adults in home settings.

#### 1) Respiratory Rate Measurements Using the Wi-Fi CSI Sensor Only

While 8 of the measurements were paired with ground-truth recordings, 11 measurements were solely taken with the unobtrusive sensor. The sample mean and standard deviation of these recordings are $12.59 \pm 3.63$ BrPM, which demonstrate a lower mean and standard deviation compared to the paired recordings of the sample population. The population averages reported in the previous literature for 749 enrolled patients over 60 years of age measured RR at rest $16.1\pm 4.28$ BrPM [75]. Although our sample number within the population is limited, the observed differences in mean and standard deviation are minor and could be attributed to individual differences and sample size.

### B. Heart Rate Measurements: Results of User Trials

The descriptive statistics of the eight heart rate (HR) measurements obtained from the CSI Wi-Fi sensor, obtained from seven volunteers, are summarized in Table 2.

**TABLE 2:** Wi-Fi sensor HR measurements against ground truth

	PPG Sensor (HR)	Wi-Fi Sensor (HR)
$Mean$	64.63 BPM	70.99 BPM
$SD$	6.35 BPM	10.52 BPM
$MAE$	9.26 BPM	
$RMSE$	9.85 BPM	
$PCC$	0.647	
$Accuracy$	85.41%	

The Wi-Fi CSI measurements yielded an average HR of $70.99 \pm 10.52$ BPM, compared to the average HR of $64.63 \pm 6.35$ BPM obtained from the ground-truth PPG sensor. Although the observed differences were minimal, the uncertainty in the measurements was considerably high in home environments. The MAE between the measurements was determined to be 9.26 BPM, while the RMSE is 9.85 BPM. Although our previous study did not include HR measurements and their accuracy metrics, it is noted that the measurement error is more significant for HR measurements than for RR. The measurement accuracy obtained is 85.41%, while the PCC between the HR measurements is 0.647, indicating a strong positive linear relationship. It is observed that HR measurement accuracy decreases in varied home environments, primarily due to high measurement variability.

The elevated HR measurement error can be attributed to the complexity of extracting HR signals from real home environments, specifically due to the nature of the minute chest movements resulting from cardiac systole and diastole. Futhermore, Wi-Fi CSI HR signals are more susceptible to noise due to body movement, environmental conditions, and individual variability. Despite the relatively low accuracy obtained for the HR signal at 83.51%, it is worth noting that the HR measurements were conducted under unconstrained and uncontrolled conditions, with transceiver distances varied between 1 and 5 meters. Although the volunteers were positioned closer to the transceivers than the researchers conducting the experiments, the presence of multiple persons may have degraded the quality of the extracted HR signal. Considering the unobtrusive nature of Wi-Fi CSI HR monitoring, the results demonstrate promising potential for further development aimed at improving measurement accuracy in various care settings for older adults.

**FIGURE 8.**
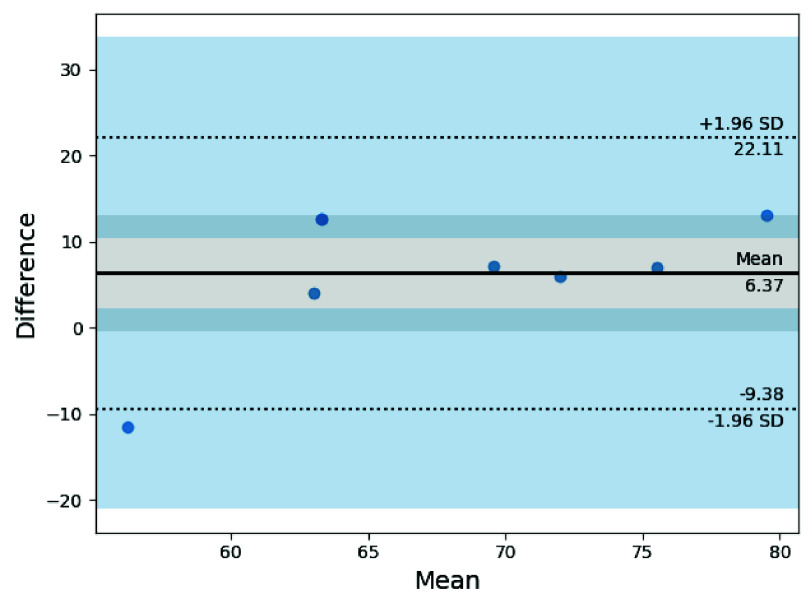
The Bland-Altman mean-difference plot for Wi-Fi CSI HR measurements against the ground-truth PPG sensor. The results indicate a positive bias and suggest homoscedasticity.

Since the captured HR measurements exhibit a wide range, Bland-Altman analysis was performed to examine the measurement agreement between our Wi-Fi CSI sensing system and the ground-truth PPG sensor. Due to the limited number of volunteer measurements, the resulting limits of agreement may be inflated and should be interpreted cautiously. Healthcare professionals must predefine the acceptable limits of agreement for HR measurements to determine whether the system’s usage is recommended for in-home monitoring. Fig. 8 presents the Bland-Altman plot between the two devices.

The Bland-Altman analysis reveals a mean difference of + 6.37 BPM between the HR measurements obtained from the Wi-Fi CSI sensor and those from the ground-truth PPG sensor, suggesting a positive bias wherein the Wi-Fi sensing system tends to overestimate heart rate values. Moreover, the limits of agreement are $[-9.38, 22.11]$ BPM, while the majority of the mean differences of the HR measurements lie within $[{0, 10}]$ BPM. This finding indicates a substantial bias between the HR measurements. Based on the range of the ground-truth values, which spans from $[{57,73}]$ BPM, the scatter of the Bland-Altman plot appears to be mostly homoscedastic, as the differences spread evenly around the mean.

Furthermore, we note that among the eight measurements taken, one data point fell outside the limits of agreement. The proportion of data points that fall within the limits of agreement must be assigned *a priori* by care professionals to determine measurement acceptability. Caution is advised when interpreting the results of this exploratory analysis, as the number of volunteers at this stage is limited. With $n=8$ paired HR measurements, the observed effect size using Cohen’s d was $d= 0.79$. At $\alpha =0.05$ with a two-tailed paired t-test, the study achieved 48.9% power, indicating that the current study is under-powered; with $n\approx 15$ number of paired measurements needed to achieve 80% power for this effect size.

It is recommended that the user trials be extended to include at least 15 older adult volunteers to draw a more comprehensive conclusion. We acknowledge that, due to the study being underpowered, we cannot confirm the observed heteroscedasticity. From the paired measurements, the Bland-Altman analysis did not reveal proportional bias in the mean difference of measurements, indicating consistent variations between observed HR values, where the measurement errors do not depend on the magnitude of the HR.

Compared to existing findings in the literature, the degradation of HR estimation performance using Wi-Fi CSI can be ascribed to the use of omnidirectional antennas. Recent studies have reported an improved accuracy of HR estimation by employing directional antennas [17]. This improvement arises from the fact that subtle chest movements due to cardiac activity are not large enough for accurate detection when using omnidirectional antennas. The authors in [17] and [76] suggest the use of multi-antenna arrays to improve measurement accuracy, where additional hardware would be needed to synchronize the antennas for sensing applications. Although the ESP32 can support an external antenna, as well as an antenna array of up to 16 antennas, it can have only manage one TX/RX path at any given time. This limitation is significant and should be taken into consideration when using the ESP32 with external antenna arrays [77]. This performance gap highlights the importance of hardware design and signal processing techniques in enhancing the accuracy and reliability of vital sign sensing in uncontrolled settings using Wi-Fi CSI.

#### 1) Heart Rate Measurements Using the Wi-Fi CSI Sensor Only

Since 11 HR measurements were not paired with the ground-truth measurements, and been analyzed separately. The mean and standard deviation of these measurements are $70.9 \pm 11.92$ BPM, which indicates a similar mean value and a higher but comparable standard deviation relative to the paired recordings of the sample population. In terms of population averages reported in previous literature, 749 enrolled patients over 60 years of age recorded their resting HR $76.3\pm 12.3$ BPM [75]. Our analysis indicates that the sample population included in our study experienced a lower average resting HR in comparison to population averages, thus would benefit from increasing the sample size to mitigate bias in measurements.

## V. Clinical and Translational Impact

The study findings suggest several clinical implications of non-contact Wi-Fi CSI vital sign monitoring for older people. While RR measurements have demonstrated promising accuracy and agreement in uncontrolled environments, HR measurements experience high variability in these scenarios due to the challenging nature of its signal extraction. The study outcomes conclude that our Wi-Fi CSI sensing system could provide valuable insights for in-home care settings in terms of RR measurements. Furthermore, it recommends algorithmic and hardware enhancements for HR monitoring. Additional research is needed to address technical limitations, such as the use of directional antennas for HR extraction. Moreover, it is essential to run several rounds of volunteer recruitment to achieve sufficient effect size and study power, thereby improving the clinical acceptability of the Wi-Fi CSI vital sign measurement system.

This study provides a framework for unobtrusive sensor testing and user trials involving older adults living independently. Informed consent is considered for each testing sub-component rather than the entirety of the study to promote inclusion and ensure the comfort of older participants. This work paves the way for future research to implement larger-scale in-home user trials, to providing a more reliable assessment of the measurement validity of the system’s performance in realistic care environments. This study contributes to the design of unobtrusive care technology for vital sign monitoring using off-the-shelf Wi-Fi CSI sensors. Implemented for the care of older adults unobtrusive technology could ease the discomfort and compliance issues associated with wearable sensors. Irregularities in the measured vital signs could indicate the onset of adverse health outcomes, enabling early clinical intervention.

## VI. Conclusion

This research describes the process of implementing experimental user trials with older adults aged 60 and above using an unobtrusive Wi-Fi CSI-based vital sign sensing system in their home environments. It discussed the experimental design considerations for older adults in their homes, such as user-centric sensor placement and the integration of informed consent with various experimental elements. In terms of signal processing, we further developed a Sample Entropy-based Principal Component (PC-SampEn) selection algorithm to detect the most prominent movements associated with cardiorespiratory activity, specifically respiratory rate (RR) and heart rate (HR). The measurement accuracy and agreement analysis were conducted on eight measurements from a total of seven volunteers for both respiratory rate and heart rate. Further long-term volunteer-based user trials are required to achieve a more significant effect size and study power, taking into account older adults’ reluctance towards the use of ground truth devices, and thus the collection of paired data. In addition, twelve RR and HR measurements were taken unpaired with a ground-truth device and compared with previous results in a controlled laboratory environment. While the performance of our Wi-Fi CSI sensing system for respiratory rate exhibited an expected degradation, heart rate estimates revealed greater sensitivity to environmental noise and body movements in realistic uncontrolled environments. Future research should focus on refining the hardware, such as exploring the effects and use cases for directional antennas, as well as optimizing the signal extraction procedure for heart rate sensing in realistic home environments. The findings from this study emphasize the technical challenges and significant potential of Wi-Fi CSI sensing systems to enable unobtrusive vital sign monitoring for the care of older adults at home.
